# Evolution of Lung Disease Studied by Computed Tomography in Adults with Cystic Fibrosis Treated with Elexacaftor/Tezacaftor/Ivacaftor

**DOI:** 10.3390/jimaging11040124

**Published:** 2025-04-21

**Authors:** Susana Hernández-Muñiz, Paloma Caballero, Adrián Peláez, Marta Solís-García, Carmen de Benavides, Javier Collada, Ignacio Díaz-Lorenzo, Cristina Zorzo, Rosa Mar Gómez-Punter, Rosa María Girón

**Affiliations:** 1 Radiology Department, University Hospital La Princesa, Calle Diego de Leon nº 62, 28006 Madrid, Spain; 2 Medicine Department, Universidad Autónoma, 28049 Madrid, Spain; 3 Centro Nacional de Investigaciones Cardiovasculares Carlos III (CNIC), 28029 Madrid, Spain; 4 Pneumonology Department, University Hospital La Princesa, 28006 Madrid, Spain; 5 Radiology Department, University Hospital Sanitas La Moraleja, 28050 Madrid, Spain

**Keywords:** cystic fibrosis, CFTR (cystic fibrosis transmembrane conductance regulator) modulator therapy, elexacaftor/tezacaftor/ivacaftor, chest imaging, computed tomography

## Abstract

Elexacaftor–tezacaftor–ivacaftor (ETI) has shown clinical and spirometric benefits in cystic fibrosis (CF). CT remains a vital tool for diagnosing and monitoring structural lung disease. This study aimed to assess the evolution of lung disease, as evaluated through CT, in adults with CF after at least one year of ETI treatment. This ambispective observational analysis assessed lung CT scans performed before initiating ETI and after at least one year of treatment, using the modified Bhalla scoring system. For those patients with an earlier CT scan, a pre-treatment phase analysis was performed. Epidemiological, clinical, and functional parameters were evaluated. Results: Sixty-two patients were included (35 males, median age 30.4 ± 7.87 years). After at least one year of ETI, significant improvements were observed in the global CT Bhalla score (12.2 ± 2.8 vs. 14.0 ± 2.8), peribronchial thickening (1.4 ± 0.6 vs. 1.0 ± 0.4), and mucus plugging (1.6 ± 0.7 vs. 0.8 ± 0.6) (*p* < 0.001). Spirometry parameters increased significantly: the percentage of the predicted forced expiratory volume in the first second (ppFEV_1_) increased from 66.5 ± 19.8 to 77.0 ± 20.4 (*p* = 0.005) and forced vital capacity (ppFVC) from 80.6 ± 16.4 to 91.6 ± 14.1 (*p* < 0.001). Additionally, body mass index showed a significant increase. A moderate correlation was found between the Bhalla score and spirometry results. In the pre-treatment phase (*n* = 52), mucus plugging demonstrated a significant worsening, whereas global CT score, other subscores, and spirometry did not change significantly. Conclusions: In adults with CF, after at least one year of ETI, a significant improvement in structural lung disease was achieved, as reflected by the CT Bhalla score.

## 1. Introduction

Although cystic fibrosis (CF) is a rare disease (incidence of 1/5000 newborns), it represents the most common severe autosomal recessive genetic disorder among the Caucasian population. Mutations in the gene encoding the CF transmembrane conductance regulator (CFTR) protein lead to dysfunction in chloride ion transport. Over 2000 mutations have been identified, but most people with CF (PwCF) harbor at least one copy of the Phe508del CFTR mutation [1,2].

CF is a multisystemic and progressive disorder that affects the pulmonary, pancreatic, hepatobiliary, gastrointestinal, reproductive, and skeletal systems. Among these, advanced pulmonary involvement is the most prominent, leading to high morbidity and mortality rates and representing the primary cause of death in most patients. Impaired mucociliary clearance results in chronic bronchopulmonary infections, mucus plugging, persistent airflow obstruction, and eventually bronchiectasis [3].

Lung computed tomography (CT) is the most sensitive imaging modality for detecting structural abnormalities in patients with CF. In most CF centers, low-dose radiation lung CT is routinely used alongside pulmonary function tests (PFT) to monitor disease progression [3,4].

Several CT scoring systems have been proposed to assess lung abnormalities in CF [3,5,6]. The Bhalla scoring system has been evaluated in multiple studies [4,7,8,9,10]; a modified version is routinely used in our institution as part of daily clinical practice. This scoring system provides a straightforward method for visually quantifying structural lung changes in CF, including bronchiectasis, mucus plugging, bronchial wall thickness, parenchymal changes, and air trapping.

In recent years, advancements in the management of CF have contributed to improved survival rates [1,11]. The development of new therapeutic approaches is having a significant impact in the treatment of PwCF. CFTR modulators are mutation-specific small molecules, including potentiators (such as ivacaftor, which facilitates channel opening) and correctors (such as lumacaftor, tezacaftor, and elexacaftor, which bind to defective CFTR proteins and partially restore their function) [11].

The triple combination therapy of elexacaftor–tezacaftor–ivacaftor (ETI) has been available in Spain since December 2021 for treatment of PwCF with at least one Phe508del mutation, who represent over 70% of the Spanish CF population [12].

While previous publications, including clinical trials and real-life studies, have demonstrated the clinical benefit of ETI in CF [13,14,15], fewer publications have focused on the evaluation of lung changes through CT scores in adult patients [16,17].

The aim of our study was to evaluate the evolution of lung disease in adult PwCF after at least one year of ETI treatment using the modified Bhalla CT score. Secondary objectives included comparing CT findings after ETI treatment with those observed in the pre-treatment phase, correlating imaging changes with PFT and body mass index (BMI), and exploring clinical and epidemiological factors that may influence structural lung changes.

## 2. Materials and Methods

### 2.1. Study Population

We conducted a prospective, observational, longitudinal study from December 2021 to February 2024, involving adults from a tertiary hospital CF unit who were receiving ETI. All participants had initiated ETI treatment before November 2022 and completed at least one year of therapy. Each patient underwent clinical, functional, and radiological follow-up.

In addition, a retrospective study was performed using data from the same cohort during the pre-treatment phase.

The study was approved by the institutional review board and ethics committee of our institution. Informed consent was obtained from all participants.

The inclusion criteria were as follows: adults aged 18 years or older with a confirmed diagnosis of CF, either heterozygous or homozygous for the Phe508del mutation, who had initiated ETI before November 2022 on the recommendation of the responsible pulmonologist and who had valid prior PFT and lung CT scans for diagnostic evaluation. Exclusion criteria were as follows: unavailable CT scans or PFT previous to ETI initiation; CT scans deemed invalid for diagnosis (due to poor technical quality or obtained during an exacerbation phase). Those patients who did not complete one year of ETI, due to adverse events or other reasons, were also excluded from the analysis.

A follow-up CT scan and PFT were scheduled for every PwCF who met the inclusion criteria, after at least 12 months of treatment. The study period ended in February 2024.

The main variable was the evaluation of the Bhalla score on CT, comparing the lung CT scans before (CT0) and after ETI (CT1). Additionally, those patients with an available CT scan prior to CT0 (referred to as CT-1) were analyzed (both overall and in separate subgroups depending on whether or not they had received prior modulator treatment).

### 2.2. Clinical and Functional Data Collection

Patients were managed by an experienced pulmonologist at our institution, which is recognized as a specialized CF center. PwCF who had been prescribed ETI were selected for inclusion in the study.

Before initiating treatment, all participants underwent a baseline clinical assessment in accordance with the standard protocols of our CF unit. Epidemiological, microbiological, and respiratory data were collected, including age, sex, genetic mutations, main microbiological pathogens, antibiotic therapy (oral or intravenous) in the previous year, BMI, and PFT.

Spirometry was performed according to current published standards [18], within a time window of 40 days before or after the corresponding CT scan. Parameters recorded included forced vital capacity (FVC), forced expiratory volume in the first second (FEV_1_), and FEV_1_/FVC ratio. These values were expressed as percentages of the predicted values (ppFVC and ppFEV_1_).

### 2.3. CT Examinations and Analysis

All PwCF underwent a chest CT scan before starting ETI treatment (CT0) and a follow-up scan was performed after at least 12 months of continuous treatment (CT1). When available, additional CT scans obtained prior to CT0 (CT-1) were also analyzed.

Volumetric, low-dose, non-contrast lung CT scans were acquired during breath-hold at full inspiration, covering the area from the pulmonary apex to the diaphragm. In addition, three to four slices were obtained at the upper, middle, and basal lung regions during maximum forced expiration. Scans were acquired using three different multi-slice CT systems: Toshiba-Canon 64-detector (Otawara, Japan), General Electric 64-detector (Chicago, IL, USA), and Philips 128-detector (Best, The Netherlands) CT. Images were reconstructed with a slice thickness of 0.625–1.5 mm.

CT images were evaluated using the modified Bhalla score by a thoracic radiologist with over 15 years of experience. Scoring was performed in batches in random order. The Bhalla score assesses the lungs as a whole, evaluating the following parameters: extent and severity of bronchiectasis, peribronchial thickening, mucus plugging, abscesses/sacculations, generations of bronchial division involved, bullae, and consolidation/collapse [5]. The modified version also includes the assessment of air trapping on expiratory images (Appendix A, Table A1). The global score is calculated by subtracting the sum of the nine individual item scores from 25 points, with higher global scores indicating better structural lung status.

To assess reliability, CT1 images were independently scored by two senior thoracic radiologists to determine inter-observer agreement. Several months later, one of them reevaluated the same images to assess intra-observer agreement.

### 2.4. Statistical Analysis

A descriptive analysis of the cohort was performed, with mean and standard deviations calculated for quantitative variables. The normality of continuous variables was assessed using the Shapiro–Wilk and Kolmogorov–Smirnov tests, while homoscedasticity was evaluated with Levene’s test. When data met the assumptions of normality and homoscedastic, parametric tests (ANOVA, t-test) were applied. In cases where these assumptions were not met, non-parametric tests (Kruskal–Wallis, Mann–Whitney, or Wilcoxon tests) were used. For qualitative variables, proportions were compared using the chi-square test or Fisher’s exact test, when appropriate. CT scan scores were assessed using Spearman’s rank correlation coefficient.

Intra- and inter-observer agreements were calculated using Cohen’s kappa indices: values from 0.00 to 0.20 were classified as “slight”, 0.21 to 0.40 as “fair”, 0.41 to 0.60 as “moderate”, 0.61 to 0.80 as “substantial”, and 0.81 or greater as “perfect.” In line with Fleiss’ interpretation, a kappa value of 0.41 or higher was considered acceptable for intra-observer agreement [19].

An ordinal logistic regression model was performed, with the dependent variable being the evolution of the global Bhalla score, which was categorized into four levels based on the change observed after ETI treatment (no change or deterioration ≤0, an increase of 1 point, an increase of 2 points, or an increase of 3 or more points). To ensure optimal model performance and convergence, a stepwise variable selection approach was applied, incorporating all relevant sociodemographic and clinical variables.

Statistical significance was defined as an alpha level of <0.05, and a *p*-value of <0.05 was considered statistically significant in all analysis. Data management, statistical analysis, and graphical representations were performed using the R statistical software, version 4.3.1.

## 3. Results

### 3.1. Baseline Characteristics of the Study Population

**Figure 1:**
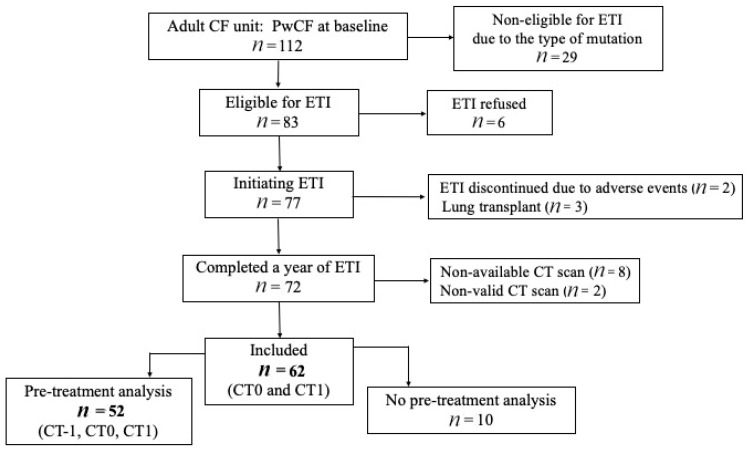
Study flow chart. CF: cystic fibrosis. ETI: elexacaftor–tezacaftor–ivacaftor. PwCF: people with cystic fibrosis.

Of the 112 patients managed at the CF center between December 2021 and November 2022, 83 were deemed eligible for ETI by the pulmonologist. After excluding 21, a total of 62 adults with CF (35 males) met the inclusion criteria (Figure 1). The median age at the initiation of ETI was 30.4 (±7.87) years, and 54.8% harbored a heterozygous mutation. The demographic and clinical characteristics of the study cohort are summarized in Table 1.

### 3.2. Changes in Radiology and Spirometry Parameters with ETI Treatment

**Figure 2:**
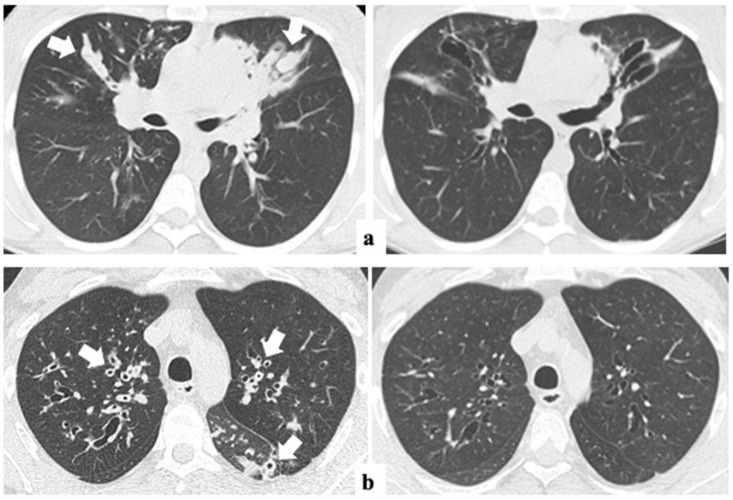
Axial lung CT images before (left) and after one-year ETI therapy (right) in two adults with CF. Dramatic improvements in mucus plugs in (**a**) (arrows) and peribronchial thickening in (**b**) (arrows) are shown. ETI: elexacaftor–tezacaftor–ivacaftor.

The median time interval between CT0 and CT1 was 2.5 ± 1.5 years (range from 12 to 54 months). The changes in the global Bhalla score and each specific structural subscore before and after ETI are summarized in Table 2. The global Bhalla score improved significantly (*p* < 0.001), from 12.2 (±2.8) to 14.0 (±2.8). Additionally, other radiological items, such as peribronchial thickening (1.4 ±0.6 vs. 1.0 ±0.4) and the extent of mucus plugs (1.6 ±0.7 vs. 0.8 ±0.6), also showed significant changes (*p* < 0.001) (Figure 2). None of the other parameters assessed worsened after one-year ETI.

Similarly, BMI (22.4 ± 2.6 vs. 23.6 ± 2.6 kg/m^2^) and spirometry (ppFEV1 66.5 ± 19.8 vs. 77.0 ± 20.4, and ppFVC 80.6 ± 16.4 vs. 91.6 ± 14.1) showed significant improvements (*p* < 0.05).

When comparing CT0 and CT1 on a per-patient basis, the global Bhalla score improved in 53 PwCF: in 16 patients, it increased by one point; in 20 patients, by two points, in 14 patients by three points; and in three patients, by more than three points. The score remained unchanged in eight patients, and worsened in only one case.

**Figure 3:**
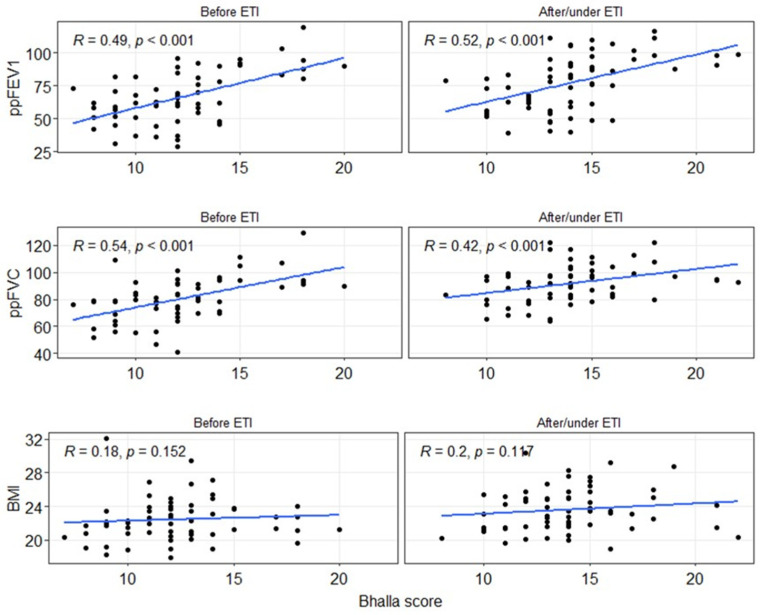
Spearman correlation between the global Bhalla score, spirometry measurements (ppFEV_1_ and ppFVC), or BMI before and after/under ETI treatment. BMI: body mass index. ETI: elexacaftor–tezacaftor–ivacaftor. ppFEV_1_: percentage of predicted forced expiratory volume in the first second. ppFVC: percentage of predicted forced vital capacity.

To assess the impact of ETI on lung function and BMI, correlation analyses were conducted between the Bhalla score, spirometry values, or BMI, both before and after ETI (Figure 3). A moderate and significant correlation (*p* < 0.001) was observed for the spirometry values, while no significant correlation (*p* > 0.05) was found for BMI. Regarding the ppFEV1 value, the correlation slightly increased after ETI (*Rho* = 0.49 to 0.52), although it remained within the moderate correlation range. In contrast, the correlation for ppFVC decreased (*Rho* = 0.54 to 0.42), but still remained within the moderate correlation range.

### 3.3. Changes in CT Scores and Spirometry Parameters in the Pre-Treatment Phase

Fifty-two of the 62 total PwCF underwent two pre-ETI scans (CT-1 and CT0). Radiological and spirometry characteristics were compared in these individuals between the beginning (CT-1) and the end (CT0) of the pre-treatment phase in order to assess their evolution in this timeframe (Table 3).

The median interval between CT-1 and CT0 was 3.2 ± 1.5 years. The global Bhalla score showed a slight, non-significant decrease (indicating a worse anatomical situation) from CT-1 (12.6 ± 2.8) to CT0 (12.1 ± 2.9), with *p* = 0.29. The extent of mucus plugs (from 1.3 ± 0.7 to 1.7 ± 0.7) was the only subscore to show significant worsening, with *p* = 0.03. Other items, such as the severity of bronchiectasis or peribronchial thickening, did not show significant worsening.

From CT-1 to CT0, the global Bhalla score remained unchanged in 21 PwCF. In 24 patients, it worsened, decreasing by one point in 12 cases, by two points in nine cases, and by more than two points in three cases. Improvement was observed in seven patients.

Spirometry values were measured in 49 PwCF during the pre-treatment phase. These values decreased from the time of CT-1 to that of CT0 (ppFEV1: 67.4 ± 19.6 vs. 65.2 ± 18.9; ppFVC: 81.9 ± 14.5 vs. 80.5 ± 16.1), although the change was not statistically significant (*p* > 0.05).

### 3.4. Impact of Previous Modulator Therapy on CT Scores in the Pre-Treatment Phase

To assess the impact of prior CFTR modulator therapy on radiological findings in the pre-treatment phase (*n* = 52), the evolution of the global Bhalla score and subscores was evaluated by categorizing patients based on whether or not they had received previous modulator therapy (Table 4).

Twenty-two PwCF had received prior modulator treatment before the initiation of ETI: tezacaftor–ivacaftor in 20 cases, ivacaftor in one case, and lumacaftor–ivacaftor in one case.

In the subgroup with prior treatment with modulators (*n* = 22), no significant changes were observed between CT-1 and CT0 in the global Bhalla score (11.5 ± 1.9 vs. 11.4 ± 2.5; *p* = 0.89) or in any of its components, including the severity of bronchiectasis (2.2 ± 0.7 vs. 2.2 ± 0.8; *p* = 0.95) and the extent of bronchiectasis (3.0 ± 0.2 in both evaluations; *p* = 0.93).

In the subgroup without prior modulator therapy (*n* = 30), while the global Bhalla score showed a non-significant worsening (13.4 ± 3.0 vs. 12.8 ± 3.0; *p* = 0.36), a significant increase was observed in the extent of mucus plugs (1.1 ± 0.8 vs. 1.6 ± 0.7; *p* = 0.02). No significant differences were detected for other Bhalla score components, such as the extent of bronchiectasis (2.8 ± 0.5 vs. 2.9 ± 0.5; *p* = 0.53), and air trapping (1.6 ± 0.6 vs. 1.5 ± 0.7; *p* = 0.99).

### 3.5. Factors Influencing the Evolution of the Bhalla Score

**Figure 4:**
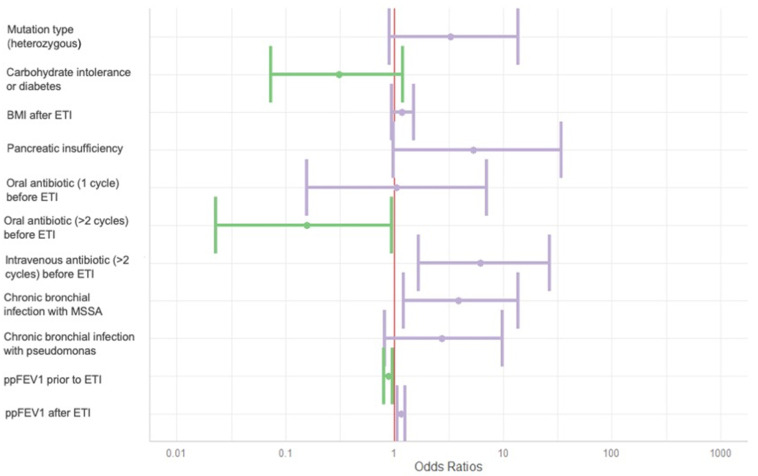
Forest plot of Bhalla score evolution predictors. BMI: body mass index. ETI: elexacaftor–tezacaftor–ivacaftor. MSSA: methicillin-sensitive *Staphilococcus aureus*. ppFEV_1_: percentage of predicted forced expiratory volume in the first second. Variables associated with a decline in the Bhalla score are shown in green, while those associated with an improvement are shown in purple.

In the multivariate analysis (Table 5 and Figure 4), the following variables were independently associated with improvement in the Bhalla score after at least one year of ETI therapy: having received more than two courses of intravenous antibiotics in the year prior to ETI initiation (OR 6.2 [1.7–26.9]); chronic bronchial infection with methicillin-sensitive *Staphylococcus aureus* (MSSA) (OR 3.9 [1.2–13.7], *p* = 0.027); and higher ppFEV1, following ETI treatment (OR 1.1 [1.1–1.3], *p* = 0.002).

Conversely, more than two courses of oral antibiotics in the year prior to ETI (OR 0.2 [0.0–0.9], *p* = 0.047), and lower ppFEV1 before ETI initiation (OR 0.9 [0.8–1.0], *p* = 0.005) were inversely associated with changes in the Bhalla score.

Other variables assessed, including mutation type, pancreatic insufficiency, and BMI, were not associated with changes in the Bhalla score over time.

### 3.6. Consistency and Reliability of Radiological Assessments

The consistency and reliability of the radiological assessments performed by different observers, and by the same observer at different times, were evaluated for the CT1 Bhalla items. Intra-observer agreement ranged from moderate to perfect, with Kappa indices of 0.968 for the global Bhalla score, 0.791 for mucus plugs, and 0.913 for peribronchial thickening. Regarding inter-observer agreement, Kappa indices varied, reflecting a range from moderate to perfect across different variables. The global Bhalla score demonstrated the highest agreement, with a Kappa index of 0.913.

## 4. Discussion

The primary finding of our study was the significant improvement in structural lung disease, as assessed by the Bhalla CT score, in adults with CF following at least one year of ETI therapy. Among the Bhalla subscores, the most marked improvements were observed in mucus plugging and peribronchial thickening. ETI treatment was also associated with improvements in PFT and BMI.

Previous studies have reported the clinical effectiveness of ETI in the CF population, highlighting improvements in lung function, respiratory symptoms, nutritional status, and quality of life. Furthermore, a reduction in the frequency of pulmonary exacerbations and sweat chloride concentration has been well described [13,14,15,20,21]. However, the impact of ETI on pulmonary structural disease assessed using CT lung scores has been addressed in only a limited number of publications to date [16,17,22,23,24]. To the best of our knowledge, ours is one of the largest series described in the literature on this topic using the modified Bhalla score.

A different scoring system, primarily the Brody II score, has been used in other publications. In a retrospective study including twelve PwCF with severe disease who received one-year ETI, Bec et al. described a significant improvement in structural lung damage on chest CT, including a 21% decrease in the visual Brody II score and a 50% reduction in mucus plugging and peribronchial thickening scores [16]. Similarly, Tagliati et al. assessed the effect of ETI on Brody CT and clinical scores in 44 PwCF after one-year treatment, reporting that the recovery of lung architecture significantly correlated with improved PFT [17].

Gushue et al. reported a reduction or complete resolution of bronchial wall thickening and mucus plugging (in 83.5% and 89% of cases, respectively) in 67 PwCF after one-year ETI. Improvements were also noted in bronchiectasis and hyperinflation, with a statistically significant reduction in mucus plugging and hyperinflation or air trapping. Additionally, better clinical outcomes, including FEV_1_, BMI, sweat chloride, and a reduction in colonization by dominant respiratory pathogens, were observed [22]. In a separate study involving 18 PwCF with advanced lung disease, a significant clinical benefit was reported after two-year compassionate use of ETI, with notable improvements in structural lung disease (using Brody score), quality of life, exacerbation rate, and BMI [23].

Recently, Cazier et al. analyzed 48 PwCF treated with ETI over the course of one year, using both the Brody score and airway size measurements. In addition to improvements in mucus plugging, airway wall thickening, and parenchymal abnormalities, they detected reversal of cylindrical bronchiectasis in a small subset of cases [24].

Our results are consistent with most of the studies mentioned above and expand upon existing literature. Unlike a few publications [10,22,24], we did not observe a reduction in the severity of bronchiectasis after one-year ETI. In accordance with previous reports, the visual CT scores in our cohort demonstrated a moderate correlation with ppFEV_1_.

In 2020, our group published a study that followed 64 PwCF without modulator therapy, observing a deterioration in structural lung condition on CT over time [4]. Prior to the introduction of ETI, several studies reported not only clinical [25] but also structural lung improvements in patients treated with ivacaftor or lumacaftor–ivacaftor [26,27,28,29,30]. Among these, Campredon et al. documented a significant decrease in mucus plugging and peribronchial thickening, but no reduction in bronchiectasis, in 283 PwCF treated with lumacaftor–ivacaftor for one year [30].

Our current study included a pre-treatment phase before the initiation of ETI, during which Bhalla CT score evolution was analyzed in 52 cases, including 22 patients who had previously received a modulator (mostly tezacaftor–ivacaftor). In the subgroup without previous modulator therapy, a significant worsening was observed in the extent of mucus plugs, although no significant changes were noted in the global score or other subscores. In contrast, no significant changes were observed in any of the subscores in the subgroup with a previous modulator. Therefore, the comparative analysis of three available CT scans for each of the 52 PwCF within the main cohort —two scans during the pre-treatment phase and one after at least one year of ETI—allowed for an extended observation period and underscored the favorable progression of the CT-assessed lung disease in PwCF treated with ETI.

This study also evaluated other parameters potentially associated with the improvement of the Bhalla CT score. Two or more courses of intravenous antibiotics and MSSA infection in the year prior to ETI initiation correlated with a higher likelihood of Bhalla score improvement. We suggest that aggressive intervention with intravenous antibiotics may better control severe pulmonary exacerbations, reducing structural lung damage and thereby enhancing the response to ETI. The positive association of Bhalla score improvement with chronic MSSA bronchial infection might reflect the lower structural impact of this microorganism compared to *Pseudomonas aeruginosa*.

Similarly, a better ppFEV_1_ post-treatment was associated with a higher likelihood of CT score improvement, whereas a lower ppFEV1 pre-treatment was linked to a reduced probability of CT score improvement. This fact reinforces the notion of a relationship between functional respiratory control and structural pulmonary changes, suggesting that more significant functional deterioration may indicate irreversible structural lung damage.

The Brody II CT scoring system [6] has been widely used in various publications to assess structural lung disease in CF. In specialized centers, more sensitive scoring systems, such as the CF-CT score (an upgraded version of Brody II) and PRAGMA-CF (Peth-Rotterdam Annotated Grid Morphometric Analysis for CF), are available [31]. At our institution, the modified Bhalla score [5] is routinely employed, as it is less complex and time-consuming than other scores, making it more practical for daily clinical practice [7,8]. Consequently, our group was very interested in studying the usefulness of this scoring system in PwCF undergoing ETI therapy.

In recent years, magnetic resonance imaging (MRI) and automated CT scoring systems have emerged for CF lung evaluation. Lung MRI has shown morphologic improvements in PwCF receiving ETI [10,32,33,34,35]. In particular, David et al. evaluated the response to ETI through the Bhalla MRI score, demonstrating the reversibility of some lung structural alterations in a significant proportion of the studied population [10]. Computer-assisted quantitative and artificial intelligence analysis of the airways and lung parenchyma offer an objective, reproducible, and streamlined approach for assessing treatment response [36,37,38,39,40]. However, these promising tools still seem to be far away for many centers.

The current study has some limitations. First, it is a single-center study including only adult patients. Second, the time lapses between the CT scans are not very homogeneous, since they were performed on the basis of routine clinical practice; the absence of standardized intervals between CT scans during the study could affect the experimental results. Finally, the use of the visual Bhalla score exclusively, and the lack of available quantitative lung CT measurements, are further limitations.

A key strength of our study is the inclusion of 52 PwCF both during the pre-treatment phase and at least one year after the onset of ETI, providing a longer-term CT assessment of lung disease evolution and enhancing the robustness of the imaging analysis.

In conclusion, our findings confirm a significant improvement in lung structural disease in adult PwCF after one-year ETI, as evidenced in the global Bhalla score, bronchial wall thickening, and mucus plugging on lung CT. Further studies are warranted to determine whether these structural improvements persist or progress beyond the first year of ETI therapy and to identify which specific subscores exhibit the most favorable trajectory over time.

## Figures and Tables

**Table 1:** Baseline characteristics of the study population. The total number of infections (*n* = 70) exceeds the number of patients included (*n* = 62) due to the presence of combined infections, most commonly *Methicillin-sensitive Staphylococcus aureus* and *Haemophilus influenza.* Data are presented as *n* (%) or mean ± standard deviation. BMI: body mass index. ppFEV_1_: percentage of predicted forced expiratory volume in the first second. ppFVC: percentage of predicted forced vital capacity.

Clinical Characteristics at the Time of Inclusion (*n* = 62)	
Sex [male]	35 (56.5%)
Age	30.4 (±7.87)
Type of mutation [heterozygous]	34 (54.8%)
Exocrine pancreas [pancreatic insufficiency]	51 (82.3%)
Endocrine pancreas [carbohydrate intolerance or diabetes]	31 (50.0%)
Hepatic disease	21 (33.9%)
ppFEV_1_	67.8 ±19.6
ppFVC	82.0 (±14.4)
BMI (kg/m^2^)	22.4 (±2.6)
Infections	
Methicillin-sensitive *Staphylococcus aureus*	36 (51.4%)
*Pseudomonas aeruginosa*	20 (28.6%)
Methicillin-resistant *Staphylococcus aureus*	3 (4.3%)
*Haemophilus influenzae*	3 (4.3%)
Non-tuberculous Mycobacterium	5 (7.1%)
Other	3 (4.3%)
Combined infections	8 (11.4%)
Prior Treatments	
Prior Modulator	25 (40.3%)
Ivacaftor	1 (1.6%)
Tezacaftor/Ivacaftor	20 (32.3%)
Both	2 (3.2%)
Lumacaftor/Ivacaftor	2 (3.2%)

**Table 2:** Changes in radiology (CT0 and CT1) and spirometry parameters with at least one-year of ETI treatment (N = 62). Data are shown as *n* (%) or mean ± standard deviation. Significant comparisons (*p* < 0.05) are highlighted in bold. BMI: body mass index. CT0: CT prior to initiation of ETI. CT1: CT after one-year ETI treatment. ETI: elexacaftor–tezacaftor–ivacaftor. ppFEV_1_: percentage of predicted forced expiratory volume in the first second. ppFVC: percentage of predicted forced vital capacity.

	Before ETI	After ETI	*p*-Value
	**CT0**	**CT1**	
**Bhalla CT score (global)**	12.2 (±2.8)	14.0 (±2.8)	**<0.001**
Severity of bronchiectasis	2.0 (±0.8)	2.0 (±0.8)	0.824
Peribronchial thickening	1.4 (±0.6)	1.0 (±0.4)	**<0.001**
Extent of the bronchiectasis	2.9 (±0.4)	2.8 (±0.5)	0.234
Extent of mucus plugs	1.6 (±0.7)	0.8 (±0.6)	**<0.001**
Abscesses or sacculations	0.2 (±0.5)	0.1 (±0.3)	0.078
Generations of bronchial division involved	2.4 (±0.6)	2.3 (±0.6)	0.306
Number of bullae	0.1 (±0.3)	0.1 (±0.3)	0.474
Collapse/consolidation	1.6 (±0.7)	1.6 (±0.6)	0.814
Air trapping	0.6 (±0.7)	0.4 (±0.7)	0.096
**Spirometry values**			
ppFEV_1_	66.5 (±19.8)	77.0 (±20.4)	**0.005**
ppFVC	80.6 (±16.4)	91.6 (±14.1)	**<0.001**
**BMI** (kg/m^2^)	22.4 (±2.6)	23.6 (±2.6)	**0.011**

**Table 3:** Changes in CT scores (CT-1 and CT0) and spirometry parameters in the pre-treatment phase (N = 52). Data are shown as *n* (%) or mean ± standard deviation. Significant comparisons (*p* <0.05) are highlighted in bold. CT0: CT prior to initiation of elexacaftor–tezacaftor–ivacaftor (ETI). CT-1: CT prior to CT0. ppFEV_1_: percentage of predicted forced expiratory volume in the first second. ppFVC: percentage of predicted forced vital capacity.

Radiology Items	CT-1	CT0	*p*-Value
Bhalla score (global)	12.6 (±2.8)	12.1 (±2.9)	0.287
Severity of bronchiectasis	1.9 (±0.8)	2.1 (±0.8)	0.382
Peribronchial thickening	1.3 (±0.6)	1.4 (±0.6)	0.406
Extent of the bronchiectasis	2.9 (±0.4)	2.9 (±0.4)	0.759
Extent of mucus plugs	1.3 (±0.7)	1.7 (±0.7)	**0.027**
Abscesses or sacculations	0.2 (±0.4)	0.2 (±0.5)	0.767
Generations of bronchial division involved	2.3 (±0.6)	2.4 (±0.6)	0.718
Number of bullae	0.1 (±0.4)	0.1 (±0.3)	0.246
Collapse/consolidation	0.6 (±0.7)	0.6 (±0.7)	0.793
Air trapping	1.7 (±0.5)	1.6 (±0.6)	0.597
**Spirometry values**			
ppFEV_1_	67.8 (±19.6)	65.8 (±18.7)	0.627
ppFVC	82.0 (±14.4)	80.7 (±15.8)	0.670

**Table 4:** Impact of previous modulator therapy on CT scores (CT-1 and CT0) in the pre-treatment phase (N = 52). Data are shown as *n* (%) or mean ± standard deviation. Significant comparisons (*p* < 0.05) are highlighted in bold. CT0: CT prior to initiation of elexacaftor–tezacaftor–ivacaftor (ETI). CT-1: CT prior to CT0.

With Prior Modulator (N = 22)	CT-1	CT0	*p*-Value
Bhalla score (global)	11.5 (±1.9)	11.4 (±2.5)	0.886
Severity of bronchiectasis	2.2 (±0.7)	2.2 (±0.8)	0.954
Peribronchial thickening	1.5 (±0.6)	1.6 (±0.6)	0.489
Extent of the bronchiectasis	3.0 (±0.2)	3.0 (±0.2)	0.927
Extent of mucus plugs	1.6 (±0.5)	1.7 (±0.8)	0.759
Abscesses or sacculations	0.2 (±0.4)	0.2 (±0.4)	0.630
Generations of bronchial division involved	2.6 (±0.5)	2.5 (±0.5)	0.863
Number of bullae	0.1 (±0.5)	0.1 (±0.4)	0.895
Collapse/consolidation	0.5 (±0.6)	0.6 (±0.6)	0.624
Air trapping	1.9 (±0.3)	1.7 (±0.6)	0.270
**Without Prior Modulator** **(N = 30)**	**CT-1**	**CT0**	***p*-Value**
Bhalla score (global)	13.4 (±3.0)	12.8 (±3.0)	0.361
Severity of bronchiectasis	1.7 (±0.8)	1.9 (±0.8)	0.424
Peribronchial thickening	1.2 (±0.5)	1.2 (±0.5)	0.859
Extent of the bronchiectasis	2.8 (±0.5)	2.9 (±0.5)	0.529
Extent of mucus plugs	1.1 (±0.8)	1.6 (±0.7)	**0.019**
Abscesses or sacculations	0.2 (±0.4)	0.2 (±0.5)	0.776
Generations of bronchial division involved	2.2 (±0.6)	2.3 (±0.6)	0.271
Number of bullae	0.1 (±0.4)	0.0 (±0.2)	0.208
Collapse/consolidation	0.6 (±0.7)	0.6 (±0.7)	0.653
Air trapping	1.6 (±0.6)	1.5 (±0.7)	0.994

**Table 5:** Factors influencing the evolution of the Bhalla score (univariate and multivariate analysis of variables associated with Bhalla score changes). Significant differences (*p* < 0.05) are indicated by bold text. BMI: body mass index. ETI: elexacaftor–tezacaftor–ivacaftor. ppFEV_1_: percentage of predicted forced expiratory volume in the first second. ppFVC: percentage of predicted forced vital capacity. MSSA: methicillin-sensitive *Staphilococcus aureus.* OR: odds ratio. CI: confidence interval.

Item	Univariate		Multivariate	
	OR [CI 95%]	*p*-Value	OR [CI 95%]	*p*-Value
Mutation type [heterozygous]	1.4 [0.5–3.7]	0.513	3.3 [0.9–13.7]	0.081
Endocrine pancreas [carbohydrate intolerance or diabetes]	1.1 [0.4–2.8]	0.864	0.3 [0.1–1.2]	0.098
BMI after ETI	1.0 [0.8–1.2]	0.927	1.2 [0.9–1.5]	0.151
Exocrine pancreas [pancreatic insufficiency]	2.6 [0.7–11]	0.171	5.3 [1.0–34.2]	0.062
Oral antibiotic [1 cycle] before ETI	1.4 [0.3–0.2]	0.647	1.0 [0.2–7.1]	0.964
Oral antibiotic [>2 cycles] before ETI	0.6 [0.3–0.2]	0.519	0.2 [0.0–0.9]	**0.047**
Intravenous antibiotic [>2 cycles] before ETI	1.6 [0.6–4.4]	0.349	6.2 [1.7–26.9]	**0.010**
Chronic bronchial infection with MSSA	2.2 [0.8–5.9]	0.122	3.9 [1.2–13.7]	**0.027**
Chronic bronchial infection with *Pseudomonas*	1.0 [0.3–2.6]	0.928	2.8 [0.8–9.9]	0.108
ppFEV_1_ prior to ETI	1.0 [1.0–1.0]	0.914	0.9 [0.8–1.0]	**0.005**
ppFEV_1_ after ETI	1.0 [1.0–1.0]	0.194	1.1 [1.1–1.3]	**0.002**

## Data Availability

The original contributions presented in this study are included in the article. Further inquiries can be directed to the corresponding author.

## Appendix A

**Table A1:** Modified Bhalla CT score.

Category	0	1	2	3
Severity of bronchiectasis	Absent	Mild (luminal diameter slightly greater than diameter of adjacent vessel)	Moderate (lumen 2 to 3 times the diameter of adjacent vessel)	Severe (lumen > 3 times the diameter of adjacent vessel)
Peribronchial thickening	Absent	Mild (wall thickness equal to the diameter of adjacent vessel)	Moderate (wall thickness greater than and up to twice the diameter of adjacent vessel)	Severe (wall thickness > 2 times the diameter of the adjacent vessel)
Extent of bronchiectasis (number of lung segments)	Absent	1–5	6–9	>9
Extent of mucus plugs (number of lung segments)	Absent	1–5	6–9	>9
Abscesses or sacculations (number of lung segments)	Absent	1–5	6–9	>9
Generations of bronchial division involved (bronchiectasis/plugging)	Absent	Up to 4th generation	Up to 5th generation	Up to 6th generation and distal
Number of bullae	Absent	Unilateral (≤4)	Bilateral (≤4)	>4
Collapse/consolidation	Absent	Subsegmental	Segmental/lobar	
Air trapping (number of lung segments)	Absent	1–5	>5	

The Bhalla score evaluates the lungs as a whole, assessing the following parameters: extent and severity of bronchiectasis, peribronchial thickening, mucus plugging, abscesses/sacculations, generations of bronchial division involved, bullae, and consolidation/collapse. The modified version also includes the assessment of air trapping on expiratory images. The global score is calculated by subtracting the sum of the individual item scores from 25 points. Therefore, the maximum possible global score is 25, reflecting a normal structural lung status; the lower the global score, the worse the anatomic status.
